# Prostate cancer and diabetes: A retrospective analysis of mortality trends in the United States (1999–2024)

**DOI:** 10.1097/MD.0000000000049267

**Published:** 2026-06-19

**Authors:** Sarim Hassan Shahab, Faizan E Mustafa, Hadia Ghazala Masood, Siddique Ahmed, Fariha Ali, Yazdan Sajid, Abubakar Nazir, Muddassir Khalid

**Affiliations:** a Nishtar Medical University, Multan, Pakistan; b Mohtarma Benazir Bhutto Shaheed Medical College, Mirpur, AJK, Pakistan; c The Jewish Hospital – Mercy Health, Cincinnati, OH.

**Keywords:** CDC, diabetes, mortality trends, United States, prostate cancer

## Abstract

Diabetes mellitus is a prevalent comorbid condition that can influence the progression of prostate cancer, which remains one of the leading malignancies among men in the United States. Knowing the long-term mortality trends in this high-risk population is essential for developing care, early diagnosis, and prevention plans. Using surveillance data from the Centers for Disease Control and Prevention, we evaluated how prostate cancer mortality among diabetic United States adults has changed over the 25 years between 1999 and 2024. International Classification of Diseases, 10th Revision codes for prostate cancer (C61) and diabetes (E10–E14) were utilized to examine mortality trends. Deaths were counted when prostate cancer was listed as the underlying cause and diabetes was listed as a contributing cause of death. We analyzed race, urban/rural, state, and census region-wise mortality trends. Joinpoint (V5.4.0) was used to analyze trends. Across 1999 to 2024, male age-adjusted mortality rates showed a fluctuating but overall stable pattern, with an initial significant decline, a marked rise after 2017, and a subsequent nonsignificant decrease, resulting in no significant long-term trend (average annual percent change = −0.41). Racial disparities were consistent, with non-Hispanic (NH) Black individuals experiencing the highest mortality rates, followed by NH Whites and Hispanics, while other groups remained stable. Urban and rural trends showed parallel temporal fluctuations, with higher rates in rural areas but no sustained long-term differences. Considerable heterogeneity was observed across states and census regions, with the West showing a significant upward trend overall. These findings warrant better, more targeted interventions that should prevent and control the deaths of prostate cancer in diabetic adults with a focus on the NH Black or African American populations and areas that are experiencing more deaths.

## 1. Introduction

Prostate cancer remains the most prevalent solid tumor in American men and also a significant cause of cancer-related morbidity and mortality. In 2021, 236,659 new cases were reported, equivalent to a rate of 112 per 100,000 men.^[1,2]^ The latest Surveillance, Epidemiology, and End Results Program estimates show that the 5-year relative survival rate is about 98%, but it has significant variations based on the subgroup of population.^[2,3]^ Such differences reinforce the significance of enhancing early detection policies and providing fair management within the high-risk populations. Diabetes mellitus (DM) is a growing health issue of the population in the United States (US). The 2023 National Diabetes Statistics Report by the Centers for Disease Control and Prevention (CDC) approximates that there are about 38.4 million Americans or approximately 11.6% of the US population have diabetes, out of which nearly 20% have not been diagnosed yet.^[4]^ Most cases (90–95%) are of type 2 diabetes and there is always a higher rate of prevalence in men as compared to women.^[4,5]^ In addition to its immediate complications, diabetes contributes substantially to morbidity, mortality, and healthcare expenditures.^[5]^ The relationship between prostate cancer and diabetes is rather complex and, to some extent, contradictory. As per the results of meta analyses, diabetes among men is linked with a modest reduction in prostate cancer risk, with pooled relative risks ranging from 0.84 to 0.86.^[6–8]^ This negative association might be an indication of biological processes like reduced levels of insulin in the blood and testosterone in diabetes.

There is also a part of detection bias because diabetic men usually have lower levels of prostate-specific antigen and are screened on lower frequencies.^[9]^ Information about results in men with both conditions has not been conclusive: even as some of them suggest a possible association between the metformin treatment and reduced risks of progression or mortality, others have found no significant association survival outcome improvement.^[10,11]^

It is based on this context that our research uses the nationally representative CDC datasets to examine the burden of prostate cancer among adults in the US with diabetes. In particular, we will consider mortality trends over this 26-year period, stratified in terms of demographic and geographic distribution of groups.

By providing a comprehensive long-term analysis of prostate cancer in diabetic individuals, our work aims to discover disparities in mortality and to inform evidence-based strategies for cancer prevention and management in vulnerable groups. It should be noted that this study does not prove the relationship between diabetes and prostate cancer. This study is limited only to examining mortality trends involving both DM and prostate cancer.

## 2. Methods

### 2.1. Study setting and inclusion criteria

This exploratory study utilized mortality statistics compiled from death certificate records available through the CDC WONDER (Centers for Disease Control and Prevention Wide-Ranging Online Data for Epidemiological Research) database and examined the timespan from 1999 to 2024 to evaluate prostate cancer-related mortality in adults having comorbid DM from the International Statistical Classification of Diseases and Related Health Problems-10th Revision (ICD-10) as follows: with E10 to E14, representing DM, and C61, representing prostate cancer. The identical ICDs have been described in the past to identify DM and prostate cancer in administrative databases, respectively.^[12,13]^ The dataset encompasses death certificate-based information on causes of death from every US state and the District of Columbia. It has served as a primary resource in numerous prior analyses evaluating mortality trends. Deaths were identified using ICD-10 codes for prostate cancer (C61) as the underlying cause of death and DM (E10–E14) as a contributing cause of death when both conditions appeared on the same death certificate. DM cases were identified from the Multiple Cause-of-Death files, whereas prostate cancer deaths were derived from the Underlying Cause-of-Death files within the CDC WONDER database for the same query. Adults in this study were marked as male decedents aged 25 years or older. The younger age groups were not selected because of unreliable estimates as recommended by CDC. This definition is consistent with prior epidemiologic analyses that have acquired a similar threshold to represent adult groups.^[14]^ Institutional review board approval was not required for this study, as it used a deidentified, publicly available government dataset and complied with Strengthening the Reporting of Observational Studies in Epidemiology guidelines.

### 2.2. Data abstraction

The retrieved data encompassed population size, year of manifestation, topographical location of death, demographic variables, urban versus rural designation, US census region, and state. Demographic information included male gender, age group, and racial/ethnic identity. The place of death was categorized as a medical facility (including outpatient, emergency department, inpatient, dead on arrival, or unspecified status), decedent’s residence, hospice, or nursing home/long-term care setting. Race and ethnicity were classified as non-Hispanic (NH) White, NH Black or African American (AA), Hispanic or Latino, and NH other (comprising NH American Indian or Alaskan Native and NH Asian or Pacific Islander). These classifications were derived from information documented on death certificates and have been employed in previous analyses utilizing the WONDER database.^[15]^ The National Center for Health Statistics Urban–Rural Classification Scheme was used to categorize areas as urban (large metropolitan regions with populations ≥ 1 million and medium/small metropolitan areas with populations between 50,000 and 999,999) or rural (populations < 50,000), consistent with the 2013 US Census definitions.^[16]^ Geographic divisions were designated as Northeast, Midwest, South, and West, following the regional framework of the US Census Bureau.^[17]^

### 2.3. Statistical analysis

The mortality trend was assessed by determining age-adjusted mortality rates (AAMRs) per 100,000 persons from 1999 through 2024. Mortality rates were reported by male sex, calendar year, race/ethnicity, state, and urban–rural status, along with their corresponding 95% confidence intervals (CIs). State-level and urban–rural data are reported only through 2020 due to CDC WONDER limitations. AAMRs were computed by standardizing deaths to the 2000 US standard population. Two authors (FEM and SHS) extracted the data independently to avoid any error. National trends mortality over time were analyzed using the Joinpoint Regression Program (version 5.4.0.0, National Cancer Institute) to estimate the annual percent change (APC) and associated 95% CIs.^[18]^

Significant temporal shifts in AAMR were recorded through the application of log-linear regression models at points where changes occurred. Average annual percent change (AAPC)/APC/Tau CIs were calculated by using parametric method in model selection. A Monte-Carlo Permutation Test was used to analyze trends. A *P*-value of <.05 was considered indicative of statistical significance.

## 3. Results

### 3.1. Place of death

Across the study duration, a total of 44,596 deaths were recorded. Most deaths occurred at the decedent’s residence, comprising 43.9% (n = 19,277) of all reported cases. Deaths in nursing homes or long-term care facilities represented 23.1% (n = 10,130), while inpatient medical facilities accounted for 21.0% (n = 9220). Hospice facilities contributed 6.1% (n = 2662) of total deaths. A smaller proportion of deaths occurred in other settings (4.4%, n = 1940) and in outpatient facilities or emergency rooms (2.7%, n = 1182). Deaths classified as dead on arrival were uncommon, representing 0.2% (n = 89), whereas cases with unknown medical facility status and unknown place of death accounted for only 0.07% (n = 31) and 0.15% (n = 65), respectively (Supplement Table 1, Supplemental Digital Content 1).

### 3.2. Overall trends

Among males, the AAMR showed an overall declining pattern during the earlier years of the study, decreasing from 2.10 per 100,000 population in 1999 to 1.66 in 2017 (APC = −1.84*; 95% CI: −2.16 to −1.51). This downward trajectory was followed by a marked rise between 2017 and 2021, during which the AAMR increased to 2.07 in 2021 (APC = 7.60*; 95% CI: 3.06 to 12.35). Thereafter, mortality rates demonstrated a modest nonsignificant decline, reaching 1.95 per 100,000 in 2024 (APC = −2.04; 95% CI: −5.79 to 1.87).

**Figure 1.**
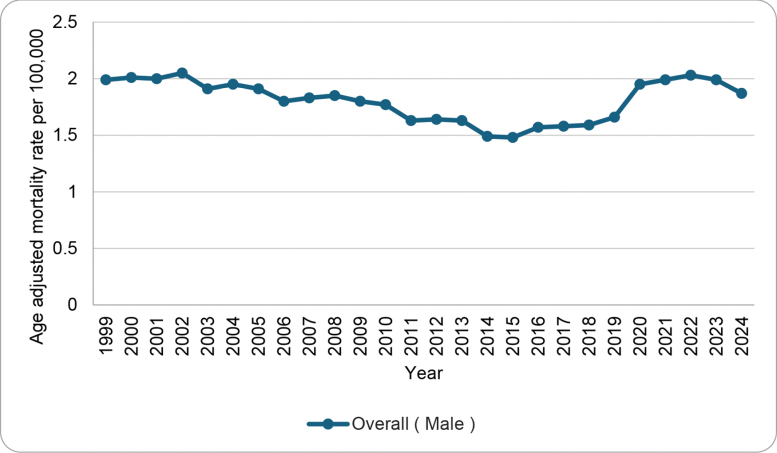
Age-adjusted mortality rate (AAMR) per 100,000 population involving prostate cancer in adults with diabetes, United States, 1999 to 2024.

Despite these temporal fluctuations, the overall trend across the entire study period from 1999 to 2024 was not statistically significant, with an AAPC of −0.41 (95% CI: −1.21 to 0.40; Fig. 1 and Supplement Table 2, Supplemental Digital Content 2).

### 3.3. Race stratified trends

Racial and ethnic disparities in mortality rates were observed throughout the study period. NH Black or AA individuals consistently demonstrated the highest AAMRs. Among this group, the AAMR declined from 2.12 per 100,000 in 1999 to 1.73 in 2011 (APC = −1.01*; 95% CI: −1.84 to −0.17). A further nonsignificant decline was noted between 2011 and 2014 (APC = −8.29; 95% CI: −20.69 to 6.05), followed by a significant upward trend from 2014 to 2024, during which the mortality rate increased to 1.78 per 100,000 in 2024 (APC = 2.71*; 95% CI: 1.70 to 3.73). However, the overall trend across the full study interval was not statistically significant (AAPC = −0.45; 95% CI: −2.14 to 1.26).

NH White individuals exhibited comparatively lower mortality rates than NH Black individuals across all years. The AAMR decreased from 0.68 per 100,000 in 1999 to 0.59 in 2017 (APC = −1.32*; 95% CI: −1.68 to −0.97). Subsequently, rates rose significantly between 2017 and 2021, peaking at 0.79 in 2021 (APC = 9.56*; 95% CI: 4.04 to 15.37), before stabilizing through 2024 at 0.75 per 100,000 (APC = −2.17; 95% CI: −6.44 to 2.31). The overall long-term trend remained nonsignificant, with an AAPC of 0.24 (95% CI: −0.71 to 1.20).

Among NH other racial groups, mortality rates remained relatively stable over the study period. The AAMR fluctuated modestly from 0.62 per 100,000 in 2000 to 0.41 in 2024, with no statistically significant temporal changes observed (APC/AAPC = −0.22; 95% CI: −1.16 to 0.73).

**Figure 2.**
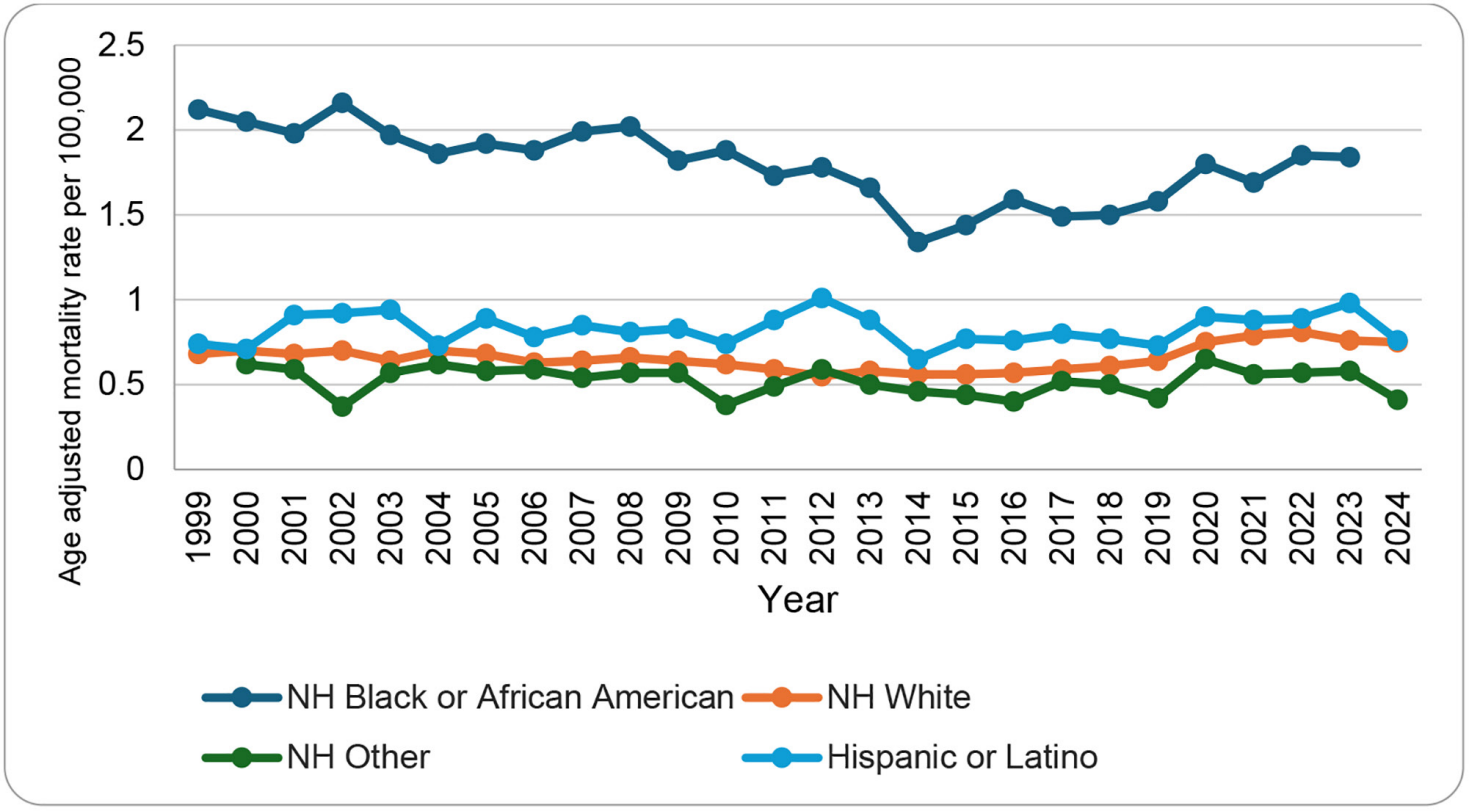
Age adjusted mortality rates per 100,000 population involving prostate cancer in adults with diabetes, stratified by race and ethnicity, United States, 1999 to 2024. NH = non-Hispanic.

Hispanic or Latino populations generally demonstrated lower mortality rates compared with NH Black individuals, with fluctuations over time but without a consistent increasing or decreasing pattern. The AAMR ranged from 0.74 per 100,000 in 1999 to 0.76 in 2024, with transient elevations observed during 2012 and 2020 to 2023. Figure 2 and Supplementary Table 3, Supplemental Digital Content 3 present the detailed race- and ethnicity-specific trends.

### 3.4. Urban/rural stratified trends

Geographic variation in mortality trends was observed between urban and rural populations. In urban (metropolitan) areas, the AAMR demonstrated a significant decline from 0.76 per 100,000 in 1999 to 0.67 in 2018 (APC = −1.17*; 95% CI: −1.47 to −0.86). This was followed by a sharp increase between 2018 and 2020, with rates rising to 0.85 per 100,000 in 2020 (APC = 15.47*; 95% CI: 4.75 to 27.28). Despite these fluctuations, the overall trend across the study period was not statistically significant (AAPC = 0.31; 95% CI: −0.59 to 1.22).

**Figure 3.**
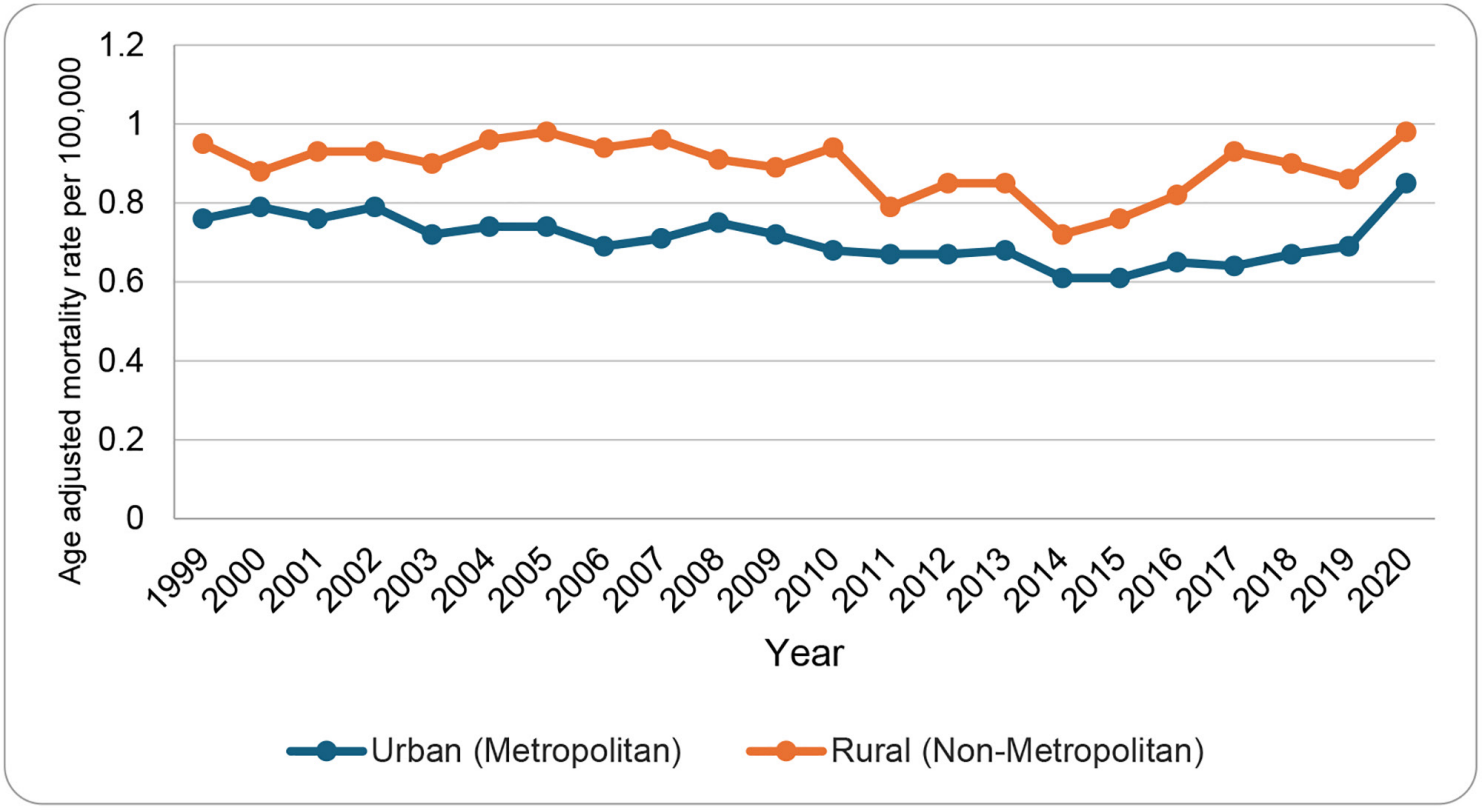
Age-adjusted mortality rate per 100,000 population involving prostate cancer mortality in adults with diabetes stratified by urban–rural classification United States, 1999 to 2020.

In rural (nonmetropolitan) regions, mortality rates were consistently higher than those observed in urban settings. The AAMR remained relatively stable between 1999 and 2007 (APC = 0.66; 95% CI: −0.93 to 2.29), followed by a significant decline from 2007 to 2014, during which rates decreased from 0.96 to 0.72 per 100,000 (APC = −3.21*; 95% CI: −5.75 to −0.60). Subsequently, mortality rates increased significantly between 2014 and 2020, reaching 0.98 per 100,000 in 2020 (APC = 3.68*; 95% CI: 1.19 to 6.23). However, the overall long-term trend for rural populations was not statistically significant (AAPC = 0.20; 95% CI: −0.97 to 1.38). Figure 3 and Supplementary Table 4, Supplemental Digital Content 4 illustrate the area-based mortality trends.

### 3.5. State stratified trends

**Figure 4.**
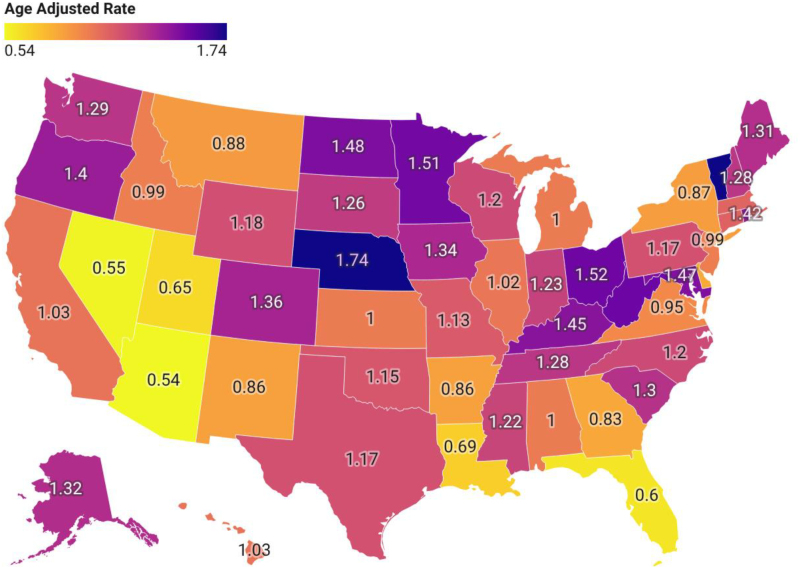
Age-adjusted mortality rates per 100,000 population involving prostate cancer in adults with diabetes stratified by state, United States, 1999 to 2020.

Marked state-level disparities in mortality rates were observed across the US. The highest AAMRs were reported in the District of Columbia (1.30; 95% CI: 1.05–1.55), Mississippi (1.21; 95% CI: 1.10–1.31), and Nebraska (1.10; 95% CI: 0.98–1.22). In contrast, the lowest AAMRs were observed in Arizona (0.41; 95% CI: 0.37–0.45), Nevada (0.44; 95% CI: 0.37–0.51), and Florida (0.47; 95% CI: 0.45–0.49). Supplementary Table 6, Supplemental Digital Content 6 summarizes the state-wise mortality distribution (Fig. 4 and Supplementary Table 5, Supplemental Digital Content 5).

### 3.6. Census region stratified trends

Regional disparities were evident across the 4 US census regions. The Northeast showed a significant long-term decline in AAMR from 0.76 in 1999 to 0.49 in 2016 (APC = −2.37*; 95% CI: −3.03 to −1.69), followed by a nonsignificant rise to 0.52 in 2024, with an overall AAPC of −1.27* (95% CI: −1.99 to −0.55).

In the Midwest, rates were stable until 2009, declined significantly to 2013 (APC = −7.91*; 95% CI: −14.26 to −1.09), then increased to 2024 (APC = 3.40*; 95% CI: 2.43 to 4.37), with no significant overall trend (AAPC = −0.03; 95% CI: −1.22 to 1.17).

The South showed an initial decline from 1999 to 2016 (APC = −1.31*; 95% CI: −1.73 to −0.87), followed by a sharp rise to 2022 (APC = 7.88*; 95% CI: 5.47 to 10.34) and a slight nonsignificant decline thereafter; overall AAPC was 0.71 (95% CI: −0.14 to 1.58).

**Figure 5.**
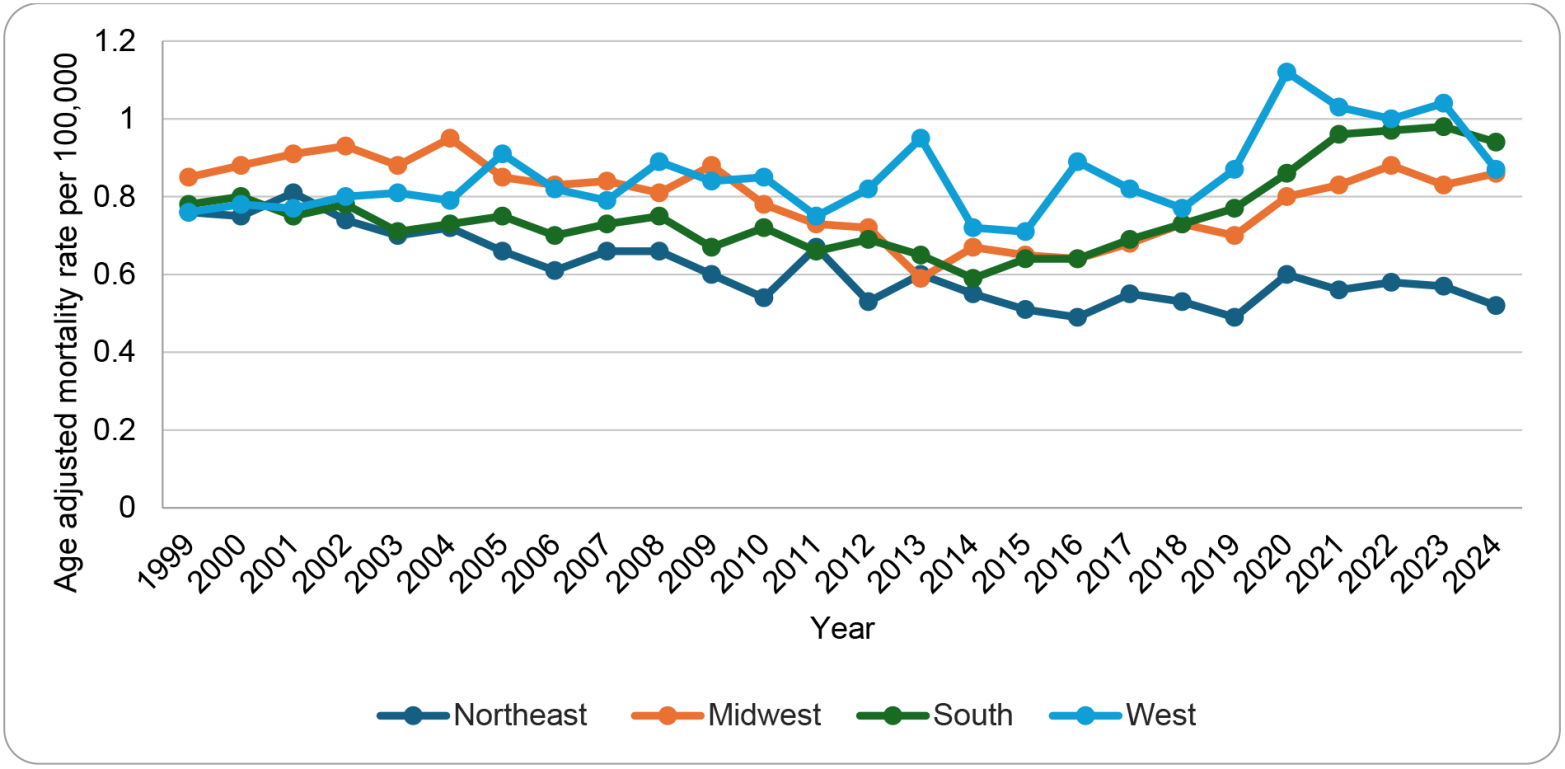
Age adjusted mortality rate (AAMR) per 100,000 population involving prostate cancer in adults with diabetes, stratified by census region, United States, 1999 to 2024.

The West demonstrated a consistent long-term increase from 0.76 to 0.87 (APC = 1.06*; 95% CI: 0.48 to 1.66), with a significant overall upward trend (AAPC = 1.06*; 95% CI: 0.48 to 1.66; Fig. 5 and Supplementary Table 6, Supplemental Digital Content 6).

## 4. Discussion

A few significant things were uncovered in this national study of prostate cancer deaths from all states that included DM as a contributing cause. First, there was a significant percentage of deaths that were not in an acute care facility, most of which were in the home, and the rest in nursing homes or inpatient facilities. The distribution also highlights that a significant proportion of patients with dual chronic disease burden (prostate cancer and diabetes) might be receiving end-of-life care in less intensive hospital settings, potentially indicative of disease progression, increased disease burden or palliative transitions or access to hospital-based care.

### 4.1. Overall temporal trends

The overall age adjusted trend in mortality for males showed a nonlinear relationship during the analysis period. The first decreases between 1999 and mid 2010s may be due to better diagnosis, treatment and management of glycemic and cardiovascular risk in diabetic patients.^[19,20]^ The observed upward and downward trends in 2017 and 2021, however, are concerning, as is the partial stabilization that has been observed. This increase could be due to a number of factors, such as expanding diabetes burden, decline in metabolic health, missed treatment due to disruption of health services, especially during the Coronavirus disease 2019 pandemic, and delayed diagnosis and treatment of cancer.^[21]^ The lack of a significant trend over the entire study period may also suggests that some progress may not have been made in recent years, and there was a high risk of vulnerability in this group.

### 4.2. Racial/ethnic differences

Consistent racial differences were found and NH Black groups had the greatest mortality rates throughout the study period. The increase from 2014 onward signals increasing or reemerging inequities, although a gradual decrease was noted in previous years.^[22]^ This pattern is probably the result of a complex mixture of biological susceptibility, increased burden of uncontrolled diabetes, and more advanced presentation of prostate cancer, as well as the misalignment in access to high-quality oncology and endocrinology services and structural inequities in health care access.^[22]^

The absolute mortality rates were lower for NH Whites, and they showed a similar trend that mortality rates declined followed by a rebound in more recent years. Hispanic populations showed more modest changes in relatively stable trends, which could be due to the “Hispanic paradox,” the differences in social support networks, the impact of migration, or the variation among Hispanic subgroups.^[23]^ There was little change among other racial groups, although the trend may be less stable due to the small numbers.

### 4.3. Urban–rural differences

The urban–rural stratification revealed a consistently higher mortality rate in rural areas than urban areas, but with similar temporal patterns. While there was a decline followed by periods of growth within the rural population, this may reflect ongoing structural barriers, including limited access to oncology professionals, endocrinology services, screening programs, and advanced treatment technologies. The differences in geographic patterns may also be related to socioeconomic factors, health insurance, transportation and the distribution of the health care workforce. In recent years, the significant increases seen in both settings also indicate a potential for stressors in the system impacting cancer and chronic disease management.^[24]^

### 4.4. Variations from state to state and region to region

There was considerable variation from state to state with the highest mortality rates occurring in areas like the District of Columbia, Mississippi, and Nebraska, and the lower rates in western states like Arizona and Nevada. Such differences may be attributable to the difference in the prevalence of diabetes, prostate cancer screening rates, health care infrastructure, socioeconomic deprivation, and state-level public health investments.^[25]^

The Northeast region showed the longest trend of decline at the census region level, which may indicate relatively high quality health systems and healthcare management of chronic diseases. The overall upward trend for the West, however, could be driven by population aging, rising diabetes rates, or changes in the population due to migration.^[26]^ Progress toward mitigating metabolic and oncologic disease burdens was somewhat unstable in the South and Midwest, with periods of improvement and resurgence.

### 4.5. Implications for clinical and public health

Prostate cancer and DM could be considered as a high-risk co-morbidity condition with complicated management issues. Diabetes can negatively impact prostate cancer in a variety of ways, such as hyperinsulinemia, chronic inflammation and metabolic imbalance, which can contribute to the progression of prostate cancer.^[6]^ Secondly, diabetes is a factor for competing cardiovascular mortality risk that can complicate treatment decisions and survivorship care.^[4]^

The results show the importance of multimodality treatment approaches that focus on both oncologic and metabolic wellbeing. Promoting integration of oncology, primary care, and endocrinology services could lead to better long-term results. Besides, specific strategies in high-risk populations, such as NH Blacks and rural residents, must be implemented to close ongoing inequities.

### 4.6. Study limitations

The results of this study are subject to some limitations that should be taken into account when interpreting the results. First, the analysis is using data from death certificates aggregated to the CDC WONDER database, so any associations between DM and prostate cancer at the individual level cannot be deduced. The results are therefore potentially vulnerable to ecological fallacy and causal inferences about the association between these conditions are not possible.

Second, multiple cause of death coding was used to determine mortality: prostate cancer as the underlying cause, and DM as a contributing cause. Such an approach relies on the accuracy and timeliness of death certificate reporting, which can differ between jurisdictions and over time, causing subject to misclassification bias, and potentially underreporting of comorbid conditions.

Third, the data are not detailed enough with respect to clinical data, such as the duration and severity of diabetes, glycemic control, the stage of cancer, or treatment modalities for diabetes or cancer in general. This meant that critical clinical and prognostic variables were not included, and therefore observed mortality trends were not directly clinically interpretable.

Fourth, mortality rates were computed using the general population as the denominator and not just those with diabetes. These estimates are not, therefore, true risk among people with diabetes with prostate cancer and temporal changes in diabetes prevalence and prostate cancer incidence could have affected observed trends.

Fifth, because of other unmeasured factors, such as obesity, smoking, socio-economic status, access to health care, screening practices, and changing treatment approaches, residual confounding is likely. Additionally, variations in prostate cancer screening (e.g., prostate-specific antigen level testing), therapeutic advances and enhancements in diabetes management during the study period may have affected mortality rates independently.

Sixth, consistency in cause-of-death classification may have been influenced by fluctuations in the coding practices of ICDs and death certification in the study period. In addition, disruptions in health care access and mortality reporting during the Coronavirus disease 2019 pandemic (especially 2020–2021) could have caused temporary variations in the observed trends.

Lastly, data for urbanization and state-level analyses were only available through 2020, making it difficult to compare subsequent years in these stratified analyses. In spite of these constraints the study does offer a descriptive picture of the national mortality trends for prostate cancer and DM over a long period.

This study is descriptive and does not prove a cause and effect relationship between death due to prostate cancer and DM. Instead, it represents population level temporal and demographic trends using multiple-cause mortality reporting.

## 5. Conclusion

The overall trends in the mortality rates among the people with prostate cancer and diabetes between 1999 and 2024 are the analyzed to point out the differences in the racial, ethnic, and geographical groups. The general mortality trend was constant yet NH Blacks were still experiencing a high mortality rate with Hispanics registering the lowest mortality rate. The disease burden of the rural areas was also high. Future research should be on the targeted public health measures and enhanced healthcare access in regions where the mortality rate remains high. The determinants of the disparity of the racial and geographical groups should also be studied. Addressing factors will reduce the dual burden of prostate cancer and diabetes.

## Author contributions

**Conceptualization:** Hadia Ghazala Masood, Siddique Ahmed.

**Data curation:** Hadia Ghazala Masood.

**Formal analysis:** Sarim Hassan Shahab, Siddique Ahmed.

**Methodology:** Siddique Ahmed, Yazdan Sajid.

**Project administration:** Sarim Hassan Shahab, Hadia Ghazala Masood, Abubakar Nazir.

**Resources:** Siddique Ahmed.

**Supervision:** Sarim Hassan Shahab, Siddique Ahmed, Abubakar Nazir.

**Validation:** Fariha Ali.

**Visualization:** Sarim Hassan Shahab, Hadia Ghazala Masood.

**Writing – original draft:** Sarim Hassan Shahab, Hadia Ghazala Masood, Siddique Ahmed, Fariha Ali, Yazdan Sajid, Muddassir Khalid.

**Writing – review & editing:** Sarim Hassan Shahab, Faizan E Mustafa, Hadia Ghazala Masood, Siddique Ahmed, Fariha Ali, Yazdan Sajid, Abubakar Nazir, Muddassir Khalid.












