# Supplementary material for: Prostate cancer and diabetes: A retrospective analysis of mortality trends in the United States (1999–2024)
Source: Medicine (Baltimore). 2026 Jun 19;105(25):e49267. doi: 10.1097/MD.0000000000049267 (PMC13286417; doi:10.1097/MD.0000000000049267)
Supplement: Supplementary file 1 [file medi-105-e49267-s001.docx]

| Place of Death | Deaths | Percentage |
| --- | --- | --- |
| Medical Facility - Inpatient | 9220 | 21.00% |
| Medical Facility - Outpatient or ER | 1182 | 2.69% |
| Medical Facility - Dead on Arrival | 89 | 0.20% |
| Medical Facility - Status unknown | 31 | 0.07% |
| Decedent's home | 19277 | 43.89% |
| Hospice facility | 2662 | 6.06% |
| Nursing home/long term care | 10130 | 23.07% |
| Other | 1940 | 4.42% |
| Place of death unknown | 65 | 0.15% |
|  | 44596 | **100%** |

**Supplementary Table 1:** Place of Death Data Table
