# Supplementary material for: Prostate cancer and diabetes: A retrospective analysis of mortality trends in the United States (1999–2024)
Source: Medicine (Baltimore). 2026 Jun 19;105(25):e49267. doi: 10.1097/MD.0000000000049267 (PMC13286417; doi:10.1097/MD.0000000000049267)
Supplement: Supplementary file 2 [file medi-105-e49267-s002.docx]

| Sex | Year | Deaths | Population | AAMR (95% CI) |
| --- | --- | --- | --- | --- |
| Male | 1999 | 1402 | 86,285,677 | 2.10 (1.99–2.21) |
| Male | 2000 | 1439 | 87,120,538 | 2.12 (2.01–2.23) |
| Male | 2001 | 1456 | 88,320,720 | 2.11 (2.00–2.22) |
| Male | 2002 | 1498 | 89,280,325 | 2.16 (2.05–2.27) |
| Male | 2003 | 1428 | 90,197,132 | 2.02 (1.91–2.12) |
| Male | 2004 | 1496 | 91,283,860 | 2.05 (1.95–2.16) |
| Male | 2005 | 1506 | 92,453,545 | 2.02 (1.91–2.12) |
| Male | 2006 | 1450 | 93,690,917 | 1.90 (1.80–1.99) |
| Male | 2007 | 1503 | 94,889,947 | 1.93 (1.83–2.03) |
| Male | 2008 | 1569 | 96,106,934 | 1.95 (1.85–2.04) |
| Male | 2009 | 1537 | 97,272,830 | 1.89 (1.80–1.99) |
| Male | 2010 | 1549 | 98,174,557 | 1.87 (1.77–1.96) |
| Male | 2011 | 1503 | 99,588,456 | 1.72 (1.63–1.81) |
| Male | 2012 | 1550 | 100,737,208 | 1.73 (1.64–1.81) |
| Male | 2013 | 1586 | 101,912,532 | 1.71 (1.63–1.80) |
| Male | 2014 | 1475 | 103,227,661 | 1.57 (1.49–1.65) |
| Male | 2015 | 1529 | 104,606,455 | 1.56 (1.48–1.64) |
| Male | 2016 | 1655 | 105,650,504 | 1.65 (1.57–1.73) |
| Male | 2017 | 1728 | 107,089,305 | 1.66 (1.58–1.74) |
| Male | 2018 | 1821 | 108,045,192 | 1.67 (1.59–1.75) |
| Male | 2019 | 1913 | 108,896,735 | 1.73 (1.66–1.81) |
| Male | 2020 | 2329 | 109,725,881 | 2.04 (1.95–2.12) |
| Male | 2021 | 2303 | 111,399,795 | 2.07 (1.99–2.16) |
| Male | 2022 | 2488 | 112,190,628 | 2.12 (2.03–2.20) |
| Male | 2023 | 2471 | 112,893,917 | 2.07 (1.99–2.15) |
| Male | 2024 | 2415 | 114,902,465 | 1.95 (1.87–2.03) |

**Supplementary Table 2:** Overall Mortality Data
