# Supplementary material for: Prostate cancer and diabetes: A retrospective analysis of mortality trends in the United States (1999–2024)
Source: Medicine (Baltimore). 2026 Jun 19;105(25):e49267. doi: 10.1097/MD.0000000000049267 (PMC13286417; doi:10.1097/MD.0000000000049267)
Supplement: Supplementary file 3 [file medi-105-e49267-s003.docx]

**Supplementary Table 3:** Race Stratified Data Table

| **Race** | **Year** | **Deaths** | **Population** | **AAMR (95% CI)** |
| --- | --- | --- | --- | --- |
| **NH Black or African American** | 1999 | 298 | 19,808,077 | 2.12 (1.88–2.36) |
| NH Black or African American | 2000 | 293 | 20,058,273 | 2.05 (1.81–2.29) |
| NH Black or African American | 2001 | 286 | 20,404,945 | 1.98 (1.75–2.21) |
| NH Black or African American | 2002 | 312 | 20,695,836 | 2.16 (1.92–2.40) |
| NH Black or African American | 2003 | 291 | 20,982,115 | 1.97 (1.74–2.19) |
| NH Black or African American | 2004 | 279 | 21,327,519 | 1.86 (1.64–2.08) |
| NH Black or African American | 2005 | 293 | 21,703,691 | 1.92 (1.69–2.14) |
| NH Black or African American | 2006 | 295 | 22,092,072 | 1.88 (1.66–2.10) |
| NH Black or African American | 2007 | 314 | 22,474,128 | 1.99 (1.76–2.21) |
| NH Black or African American | 2008 | 334 | 22,857,307 | 2.02 (1.80–2.24) |
| NH Black or African American | 2009 | 311 | 23,236,715 | 1.82 (1.61–2.02) |
| NH Black or African American | 2010 | 327 | 23,537,629 | 1.88 (1.67–2.09) |
| NH Black or African American | 2011 | 311 | 23,958,352 | 1.73 (1.53–1.92) |
| NH Black or African American | 2012 | 340 | 24,345,607 | 1.78 (1.59–1.98) |
| NH Black or African American | 2013 | 317 | 24,743,381 | 1.66 (1.48–1.85) |
| NH Black or African American | 2014 | 271 | 25,244,585 | 1.34 (1.18–1.50) |
| NH Black or African American | 2015 | 304 | 25,752,287 | 1.44 (1.28–1.61) |
| NH Black or African American | 2016 | 346 | 26,212,105 | 1.59 (1.42–1.76) |
| NH Black or African American | 2017 | 340 | 26,762,620 | 1.49 (1.33–1.65) |
| NH Black or African American | 2018 | 351 | 27,178,229 | 1.50 (1.34–1.66) |
| NH Black or African American | 2019 | 382 | 27,592,123 | 1.58 (1.42–1.75) |
| NH Black or African American | 2020 | 450 | 27,969,928 | 1.80 (1.63–1.97) |
| NH Black or African American | 2021 | 429 | 27,538,737 | 1.69 (1.53–1.87) |
| NH Black or African American | 2022 | 483 | 27,685,911 | 1.85 (1.69–2.03) |
| NH Black or African American | 2023 | 483 | 28,042,483 | 1.84 (1.67–2.01) |
| NH Black or African American | 2024 | 495 | 28,588,970 | 1.78 (1.62–1.95) |

| **Race** | **Year** | **Deaths** | **Population** | **AAMR (95% CI)** |
| --- | --- | --- | --- | --- |
| **NH White** | 1999 | 1030 | 134,935,890 | 0.68 (0.64–0.72) |
| NH White | 2000 | 1057 | 135,202,971 | 0.70 (0.66–0.74) |
| NH White | 2001 | 1058 | 135,651,679 | 0.68 (0.64–0.72) |
| NH White | 2002 | 1084 | 136,026,843 | 0.70 (0.65–0.74) |
| NH White | 2003 | 1015 | 136,415,868 | 0.64 (0.60–0.68) |
| NH White | 2004 | 1101 | 136,956,601 | 0.70 (0.66–0.74) |
| NH White | 2005 | 1081 | 137,614,452 | 0.68 (0.64–0.72) |
| NH White | 2006 | 1029 | 138,363,022 | 0.63 (0.59–0.67) |
| NH White | 2007 | 1051 | 139,060,265 | 0.64 (0.60–0.67) |
| NH White | 2008 | 1093 | 139,772,453 | 0.66 (0.62–0.70) |
| NH White | 2009 | 1078 | 140,451,863 | 0.64 (0.60–0.68) |
| NH White | 2010 | 1087 | 140,987,505 | 0.62 (0.59–0.66) |
| NH White | 2011 | 1021 | 141,789,725 | 0.59 (0.55–0.62) |
| NH White | 2012 | 998 | 142,424,668 | 0.55 (0.52–0.59) |
| NH White | 2013 | 1072 | 143,045,234 | 0.58 (0.55–0.62) |
| NH White | 2014 | 1030 | 143,642,265 | 0.56 (0.53–0.60) |
| NH White | 2015 | 1037 | 144,347,874 | 0.56 (0.52–0.59) |
| NH White | 2016 | 1111 | 144,979,180 | 0.57 (0.54–0.61) |
| NH White | 2017 | 1159 | 145,456,222 | 0.59 (0.56–0.63) |
| NH White | 2018 | 1230 | 145,801,376 | 0.61 (0.58–0.64) |
| NH White | 2019 | 1303 | 146,079,678 | 0.64 (0.60–0.67) |
| NH White | 2020 | 1561 | 146,337,256 | 0.75 (0.71–0.79) |
| NH White | 2021 | 1566 | 144,655,323 | 0.79 (0.75–0.83) |
| NH White | 2022 | 1683 | 144,499,701 | 0.81 (0.77–0.85) |
| NH White | 2023 | 1620 | 144,415,042 | 0.76 (0.72–0.79) |
| NH White | 2024 | 1619 | 144,849,472 | 0.75 (0.71–0.78) |

| **Race** | **Year** | **Deaths** | **Population** | **AAMR (95% CI)** |
| --- | --- | --- | --- | --- |
| **Hispanic or Latino** | 1999 | 57 | 17,503,631 | 0.74 (0.55–0.96) |
| Hispanic or Latino | 2000 | 58 | 18,219,679 | 0.71 (0.54–0.93) |
| Hispanic or Latino | 2001 | 82 | 19,290,018 | 0.91 (0.72–1.14) |
| Hispanic or Latino | 2002 | 79 | 20,159,630 | 0.92 (0.73–1.16) |
| Hispanic or Latino | 2003 | 89 | 21,011,656 | 0.94 (0.75–1.16) |
| Hispanic or Latino | 2004 | 73 | 21,877,214 | 0.73 (0.57–0.92) |
| Hispanic or Latino | 2005 | 95 | 22,804,023 | 0.89 (0.72–1.10) |
| Hispanic or Latino | 2006 | 85 | 23,743,864 | 0.78 (0.62–0.97) |
| Hispanic or Latino | 2007 | 104 | 24,673,919 | 0.85 (0.68–1.02) |
| Hispanic or Latino | 2008 | 99 | 25,602,850 | 0.81 (0.65–0.99) |
| Hispanic or Latino | 2009 | 106 | 26,504,021 | 0.83 (0.66–0.99) |
| Hispanic or Latino | 2010 | 101 | 27,192,663 | 0.74 (0.59–0.88) |
| Hispanic or Latino | 2011 | 126 | 28,255,675 | 0.88 (0.72–1.03) |
| Hispanic or Latino | 2012 | 152 | 28,988,437 | 1.01 (0.84–1.17) |
| Hispanic or Latino | 2013 | 147 | 29,784,174 | 0.88 (0.73–1.02) |
| Hispanic or Latino | 2014 | 119 | 30,809,714 | 0.65 (0.53–0.77) |
| Hispanic or Latino | 2015 | 137 | 31,761,872 | 0.77 (0.64–0.90) |
| Hispanic or Latino | 2016 | 149 | 32,438,262 | 0.76 (0.63–0.88) |
| Hispanic or Latino | 2017 | 161 | 33,594,503 | 0.80 (0.68–0.93) |
| Hispanic or Latino | 2018 | 166 | 34,350,362 | 0.77 (0.65–0.90) |
| Hispanic or Latino | 2019 | 167 | 35,025,850 | 0.73 (0.62–0.85) |
| Hispanic or Latino | 2020 | 216 | 35,758,193 | 0.90 (0.78–1.03) |
| Hispanic or Latino | 2021 | 211 | 36,687,942 | 0.88 (0.76–1.01) |
| Hispanic or Latino | 2022 | 220 | 37,391,420 | 0.89 (0.78–1.02) |
| Hispanic or Latino | 2023 | 257 | 38,599,156 | 0.98 (0.86–1.11) |
| Hispanic or Latino | 2024 | 207 | 40,491,544 | 0.76 (0.65–0.87) |

| **Race** | **Year** | **Deaths** | **Population** | **AAMR (95% CI)** |
| --- | --- | --- | --- | --- |
| **NH Other** | 2000 | 28 | 8,503,717 | 0.62 (0.41–0.90) |
| NH Other | 2001 | 26 | 8,958,486 | 0.59 (0.38–0.86) |
| NH Other | 2002 | 20 | 9,325,719 | 0.37 (0.22–0.57) |
| NH Other | 2003 | 30 | 9,680,790 | 0.57 (0.38–0.82) |
| NH Other | 2004 | 35 | 10,044,050 | 0.62 (0.43–0.87) |
| NH Other | 2005 | 36 | 10,429,218 | 0.58 (0.40–0.82) |
| NH Other | 2006 | 37 | 10,820,401 | 0.59 (0.41–0.81) |
| NH Other | 2007 | 33 | 11,195,465 | 0.54 (0.37–0.76) |
| NH Other | 2008 | 41 | 11,562,480 | 0.57 (0.41–0.78) |
| NH Other | 2009 | 42 | 11,914,417 | 0.57 (0.41–0.77) |
| NH Other | 2010 | 30 | 12,174,186 | 0.38 (0.25–0.55) |
| NH Other | 2011 | 43 | 12,589,184 | 0.49 (0.35–0.67) |
| NH Other | 2012 | 54 | 13,067,325 | 0.59 (0.44–0.78) |
| NH Other | 2013 | 48 | 13,512,525 | 0.50 (0.37–0.66) |
| NH Other | 2014 | 49 | 14,112,716 | 0.46 (0.34–0.62) |
| NH Other | 2015 | 48 | 14,691,784 | 0.44 (0.32–0.58) |
| NH Other | 2016 | 46 | 15,011,870 | 0.40 (0.29–0.53) |
| NH Other | 2017 | 65 | 15,633,986 | 0.52 (0.40–0.67) |
| NH Other | 2018 | 71 | 15,981,223 | 0.50 (0.39–0.64) |
| NH Other | 2019 | 58 | 16,283,516 | 0.42 (0.32–0.54) |
| NH Other | 2020 | 97 | 16,569,636 | 0.65 (0.53–0.80) |
| NH Other | 2021 | 80 | 16,030,238 | 0.56 (0.44–0.70) |
| NH Other | 2022 | 89 | 16,476,594 | 0.57 (0.45–0.70) |
| NH Other | 2023 | 96 | 16,894,995 | 0.58 (0.47–0.71) |
| NH Other | 2024 | 72 | 17,933,903 | 0.41 (0.32–0.52) |
