# Supplementary material for: Prostate cancer and diabetes: A retrospective analysis of mortality trends in the United States (1999–2024)
Source: Medicine (Baltimore). 2026 Jun 19;105(25):e49267. doi: 10.1097/MD.0000000000049267 (PMC13286417; doi:10.1097/MD.0000000000049267)
Supplement: Supplementary file 4 [file medi-105-e49267-s004.docx]

**Supplementary Table 4:** Urban Rural Data Table

| **Area** | **Year** | **Deaths** | **Population** | **AAMR (95% CI)** |
| --- | --- | --- | --- | --- |
| Urban (Metropolitan) | 1999 | 1078 | 151,245,342 | 0.76 (0.72–0.81) |
| Urban (Metropolitan) | 2000 | 1136 | 152,658,699 | 0.79 (0.75–0.84) |
| Urban (Metropolitan) | 2001 | 1132 | 154,896,258 | 0.76 (0.72–0.81) |
| Urban (Metropolitan) | 2002 | 1178 | 156,659,051 | 0.79 (0.74–0.83) |
| Urban (Metropolitan) | 2003 | 1110 | 158,367,715 | 0.72 (0.68–0.77) |
| Urban (Metropolitan) | 2004 | 1164 | 160,272,097 | 0.74 (0.70–0.79) |
| Urban (Metropolitan) | 2005 | 1155 | 162,371,826 | 0.74 (0.70–0.79) |
| Urban (Metropolitan) | 2006 | 1106 | 164,523,389 | 0.69 (0.65–0.73) |
| Urban (Metropolitan) | 2007 | 1153 | 166,650,886 | 0.71 (0.67–0.75) |
| Urban (Metropolitan) | 2008 | 1232 | 168,826,027 | 0.75 (0.71–0.79) |
| Urban (Metropolitan) | 2009 | 1209 | 170,965,574 | 0.72 (0.68–0.76) |
| Urban (Metropolitan) | 2010 | 1189 | 172,591,105 | 0.68 (0.64–0.72) |
| Urban (Metropolitan) | 2011 | 1195 | 175,204,532 | 0.67 (0.63–0.71) |
| Urban (Metropolitan) | 2012 | 1219 | 177,423,676 | 0.67 (0.63–0.70) |
| Urban (Metropolitan) | 2013 | 1253 | 179,634,449 | 0.68 (0.64–0.72) |
| Urban (Metropolitan) | 2014 | 1179 | 182,304,016 | 0.61 (0.57–0.64) |
| Urban (Metropolitan) | 2015 | 1209 | 184,959,306 | 0.61 (0.58–0.65) |
| Urban (Metropolitan) | 2016 | 1313 | 186,963,190 | 0.65 (0.61–0.68) |
| Urban (Metropolitan) | 2017 | 1340 | 189,675,062 | 0.64 (0.60–0.67) |
| Urban (Metropolitan) | 2018 | 1433 | 191,415,024 | 0.67 (0.63–0.70) |
| Urban (Metropolitan) | 2019 | 1525 | 193,006,488 | 0.69 (0.66–0.73) |
| Urban (Metropolitan) | 2020 | 1887 | 194,600,110 | 0.85 (0.81–0.88) |
| Rural (Non-Metropolitan) | 1999 | 324 | 29,163,427 | 0.95 (0.85–1.05) |
| Rural (Non-Metropolitan) | 2000 | 303 | 29,325,941 | 0.88 (0.78–0.98) |
| Rural (Non-Metropolitan) | 2001 | 324 | 29,408,870 | 0.93 (0.83–1.03) |
| Rural (Non-Metropolitan) | 2002 | 320 | 29,548,977 | 0.93 (0.83–1.03) |
| Rural (Non-Metropolitan) | 2003 | 318 | 29,722,714 | 0.90 (0.80–1.00) |
| Rural (Non-Metropolitan) | 2004 | 332 | 29,933,287 | 0.96 (0.85–1.06) |
| Rural (Non-Metropolitan) | 2005 | 351 | 30,179,558 | 0.98 (0.88–1.08) |
| Rural (Non-Metropolitan) | 2006 | 344 | 30,495,970 | 0.94 (0.84–1.04) |
| Rural (Non-Metropolitan) | 2007 | 350 | 30,752,891 | 0.96 (0.86–1.06) |
| Rural (Non-Metropolitan) | 2008 | 337 | 30,969,063 | 0.91 (0.81–1.01) |
| Rural (Non-Metropolitan) | 2009 | 328 | 31,141,442 | 0.89 (0.79–0.99) |
| Rural (Non-Metropolitan) | 2010 | 360 | 31,300,878 | 0.94 (0.84–1.04) |
| Rural (Non-Metropolitan) | 2011 | 308 | 31,388,404 | 0.79 (0.70–0.88) |
| Rural (Non-Metropolitan) | 2012 | 331 | 31,402,361 | 0.85 (0.76–0.94) |
| Rural (Non-Metropolitan) | 2013 | 333 | 31,450,865 | 0.85 (0.76–0.94) |
| Rural (Non-Metropolitan) | 2014 | 296 | 31,505,264 | 0.72 (0.64–0.80) |
| Rural (Non-Metropolitan) | 2015 | 320 | 31,594,511 | 0.76 (0.68–0.85) |
| Rural (Non-Metropolitan) | 2016 | 342 | 31,678,227 | 0.82 (0.73–0.91) |
| Rural (Non-Metropolitan) | 2017 | 388 | 31,772,269 | 0.93 (0.83–1.02) |
| Rural (Non-Metropolitan) | 2018 | 388 | 31,896,166 | 0.90 (0.81–0.99) |
| Rural (Non-Metropolitan) | 2019 | 388 | 31,974,679 | 0.86 (0.77–0.94) |
| Rural (Non-Metropolitan) | 2020 | 442 | 32,028,405 | 0.98 (0.89–1.07) |
