# Supplementary material for: Prostate cancer and diabetes: A retrospective analysis of mortality trends in the United States (1999–2024)
Source: Medicine (Baltimore). 2026 Jun 19;105(25):e49267. doi: 10.1097/MD.0000000000049267 (PMC13286417; doi:10.1097/MD.0000000000049267)
Supplement: Supplementary file 5 [file medi-105-e49267-s005.docx]

**Supplementary Table 5:** State Stratified Data Table

| **State** | **Deaths** | **Population** | **AAMR (95% CI)** |
| --- | --- | --- | --- |
| Alabama | 533 | 68,915,349 | 0.71 (0.65–0.77) |
| Alaska | 38 | 9,623,822 | 0.66 (0.46–0.92) |
| Arizona | 417 | 90,089,006 | 0.41 (0.37–0.45) |
| Arkansas | 307 | 41,711,420 | 0.63 (0.56–0.70) |
| California | 4854 | 530,625,638 | 0.95 (0.92–0.98) |
| Colorado | 562 | 73,189,174 | 0.88 (0.81–0.96) |
| Connecticut | 402 | 53,012,824 | 0.64 (0.58–0.71) |
| Delaware | 87 | 13,210,778 | 0.60 (0.48–0.74) |
| District of Columbia | 109 | 9,448,827 | 1.30 (1.05–1.55) |
| Florida | 1824 | 289,701,852 | 0.47 (0.45–0.49) |
| Georgia | 717 | 135,415,629 | 0.61 (0.56–0.65) |
| Hawaii | 137 | 20,066,183 | 0.58 (0.48–0.68) |
| Idaho | 183 | 21,392,071 | 0.83 (0.71–0.96) |
| Illinois | 1226 | 184,489,320 | 0.64 (0.60–0.68) |
| Indiana | 740 | 92,386,904 | 0.77 (0.71–0.82) |
| Iowa | 468 | 44,030,204 | 0.83 (0.76–0.91) |
| Kansas | 286 | 39,931,570 | 0.63 (0.55–0.70) |
| Kentucky | 535 | 63,061,451 | 0.81 (0.74–0.88) |
| Louisiana | 415 | 64,895,449 | 0.64 (0.57–0.70) |
| Maine | 186 | 20,521,512 | 0.74 (0.64–0.85) |
| Maryland | 754 | 84,902,583 | 0.90 (0.84–0.97) |
| Massachusetts | 559 | 99,096,796 | 0.50 (0.46–0.54) |
| Michigan | 1093 | 145,550,610 | 0.67 (0.63–0.71) |
| Minnesota | 838 | 77,002,440 | 1.00 (0.94–1.07) |
| Mississippi | 519 | 41,496,865 | 1.21 (1.10–1.31) |
| Missouri | 605 | 86,518,061 | 0.62 (0.57–0.67) |
| Montana | 134 | 14,577,633 | 0.78 (0.65–0.92) |
| Nebraska | 331 | 25,869,503 | 1.10 (0.98–1.22) |
| Nevada | 153 | 38,580,189 | 0.44 (0.37–0.51) |
| New Hampshire | 163 | 19,856,602 | 0.78 (0.66–0.90) |
| New Jersey | 829 | 130,947,870 | 0.57 (0.54–0.61) |
| New Mexico | 176 | 28,568,524 | 0.58 (0.50–0.67) |
| New York | 1748 | 288,624,005 | 0.56 (0.53–0.58) |
| North Carolina | 1174 | 137,233,223 | 0.84 (0.79–0.88) |
| North Dakota | 115 | 9,822,062 | 0.93 (0.76–1.11) |
| Ohio | 1785 | 169,651,360 | 0.92 (0.88–0.97) |
| Oklahoma | 553 | 53,110,132 | 0.95 (0.87–1.03) |
| Oregon | 640 | 57,214,641 | 1.01 (0.93–1.09) |
| Pennsylvania | 1746 | 189,975,429 | 0.74 (0.71–0.78) |
| Rhode Island | 122 | 15,765,032 | 0.65 (0.53–0.76) |
| South Carolina | 554 | 67,227,226 | 0.78 (0.72–0.85) |
| South Dakota | 133 | 11,603,220 | 0.93 (0.77–1.09) |
| Tennessee | 707 | 92,689,763 | 0.72 (0.67–0.77) |
| Texas | 2541 | 344,632,461 | 0.84 (0.81–0.88) |
| Utah | 227 | 34,057,911 | 0.80 (0.69–0.90) |
| Vermont | 97 | 9,393,709 | 0.92 (0.75–1.13) |
| Virginia | 761 | 116,579,933 | 0.69 (0.64–0.74) |
| Washington | 777 | 98,931,323 | 0.80 (0.74–0.86) |
| West Virginia | 275 | 28,023,276 | 0.82 (0.72–0.92) |
| Wisconsin | 720 | 82,712,057 | 0.77 (0.71–0.83) |
| Wyoming | 67 | 7,921,067 | 0.86 (0.66–1.09) |
