# Supplementary material for: Prostate cancer and diabetes: A retrospective analysis of mortality trends in the United States (1999–2024)
Source: Medicine (Baltimore). 2026 Jun 19;105(25):e49267. doi: 10.1097/MD.0000000000049267 (PMC13286417; doi:10.1097/MD.0000000000049267)
Supplement: Supplementary file 6 [file medi-105-e49267-s006.docx]

| Region | Year | Deaths | Population | AAMR (95% CI) |
| --- | --- | --- | --- | --- |
| Northeast | 1999 | 287 | 35,633,134 | 0.76 (0.67–0.85) |
| Northeast | 2000 | 287 | 35,788,687 | 0.75 (0.66–0.83) |
| Northeast | 2001 | 311 | 36,006,250 | 0.81 (0.72–0.90) |
| Northeast | 2002 | 290 | 36,185,082 | 0.74 (0.66–0.83) |
| Northeast | 2003 | 269 | 36,346,948 | 0.70 (0.61–0.78) |
| Northeast | 2004 | 285 | 36,462,699 | 0.72 (0.63–0.80) |
| Northeast | 2005 | 263 | 36,559,788 | 0.66 (0.58–0.74) |
| Northeast | 2006 | 249 | 36,682,176 | 0.61 (0.53–0.68) |
| Northeast | 2007 | 267 | 36,846,338 | 0.66 (0.58–0.74) |
| Northeast | 2008 | 271 | 37,084,149 | 0.66 (0.58–0.74) |
| Northeast | 2009 | 254 | 37,339,597 | 0.60 (0.53–0.68) |
| Northeast | 2010 | 241 | 37,543,347 | 0.54 (0.48–0.61) |
| Northeast | 2011 | 288 | 37,864,117 | 0.67 (0.59–0.75) |
| Northeast | 2012 | 237 | 38,158,527 | 0.53 (0.46–0.60) |
| Northeast | 2013 | 269 | 38,437,194 | 0.60 (0.53–0.68) |
| Northeast | 2014 | 252 | 38,710,627 | 0.55 (0.48–0.62) |
| Northeast | 2015 | 238 | 38,965,872 | 0.51 (0.44–0.58) |
| Northeast | 2016 | 225 | 39,040,202 | 0.49 (0.42–0.55) |
| Northeast | 2017 | 263 | 39,417,175 | 0.55 (0.48–0.61) |
| Northeast | 2018 | 260 | 39,321,978 | 0.53 (0.46–0.59) |
| Northeast | 2019 | 244 | 39,381,333 | 0.49 (0.43–0.55) |
| Northeast | 2020 | 302 | 39,418,559 | 0.60 (0.53–0.67) |
| Northeast | 2021 | 281 | 40,339,548 | 0.56 (0.49–0.63) |
| Northeast | 2022 | 309 | 40,289,184 | 0.58 (0.52–0.65) |
| Northeast | 2023 | 299 | 40,402,747 | 0.57 (0.51–0.64) |
| Northeast | 2024 | 282 | 41,034,160 | 0.52 (0.46–0.58) |
| Midwest | 1999 | 363 | 41,293,967 | 0.85 (0.76–0.93) |
| Midwest | 2000 | 373 | 41,504,992 | 0.88 (0.79–0.96) |
| Midwest | 2001 | 389 | 41,762,990 | 0.91 (0.82–1.00) |
| Midwest | 2002 | 408 | 41,970,731 | 0.93 (0.84–1.02) |
| Midwest | 2003 | 386 | 42,200,881 | 0.88 (0.79–0.97) |
| Midwest | 2004 | 413 | 42,455,980 | 0.95 (0.85–1.04) |
| Midwest | 2005 | 379 | 42,748,579 | 0.85 (0.77–0.94) |
| Midwest | 2006 | 381 | 43,084,311 | 0.83 (0.75–0.92) |
| Midwest | 2007 | 389 | 43,424,366 | 0.84 (0.76–0.92) |
| Midwest | 2008 | 382 | 43,718,509 | 0.81 (0.73–0.90) |
| Midwest | 2009 | 410 | 44,010,460 | 0.88 (0.79–0.96) |
| Midwest | 2010 | 382 | 44,248,465 | 0.78 (0.70–0.86) |
| Midwest | 2011 | 346 | 44,584,105 | 0.73 (0.65–0.81) |
| Midwest | 2012 | 364 | 44,817,227 | 0.72 (0.64–0.79) |
| Midwest | 2013 | 300 | 45,090,597 | 0.59 (0.52–0.66) |
| Midwest | 2014 | 343 | 45,360,409 | 0.67 (0.60–0.74) |
| Midwest | 2015 | 337 | 45,628,315 | 0.65 (0.58–0.72) |
| Midwest | 2016 | 345 | 45,802,491 | 0.64 (0.57–0.70) |
| Midwest | 2017 | 373 | 46,143,783 | 0.68 (0.61–0.75) |
| Midwest | 2018 | 411 | 46,405,110 | 0.73 (0.65–0.80) |
| Midwest | 2019 | 404 | 46,589,364 | 0.70 (0.63–0.77) |
| Midwest | 2020 | 462 | 46,721,679 | 0.80 (0.73–0.88) |
| Midwest | 2021 | 461 | 47,032,092 | 0.83 (0.75–0.91) |
| Midwest | 2022 | 520 | 47,087,294 | 0.88 (0.80–0.96) |
| Midwest | 2023 | 496 | 47,312,396 | 0.83 (0.76–0.91) |
| Midwest | 2024 | 520 | 47,893,529 | 0.86 (0.79–0.94) |
| South | 1999 | 489 | 64,108,630 | 0.78 (0.71–0.85) |
| South | 2000 | 497 | 64,843,390 | 0.80 (0.73–0.87) |
| South | 2001 | 479 | 65,874,012 | 0.75 (0.69–0.82) |
| South | 2002 | 506 | 66,758,178 | 0.78 (0.71–0.85) |
| South | 2003 | 466 | 67,639,133 | 0.71 (0.64–0.77) |
| South | 2004 | 492 | 68,718,205 | 0.73 (0.67–0.80) |
| South | 2005 | 510 | 69,951,038 | 0.75 (0.68–0.81) |
| South | 2006 | 485 | 71,198,336 | 0.70 (0.63–0.76) |
| South | 2007 | 523 | 72,387,467 | 0.73 (0.67–0.79) |
| South | 2008 | 542 | 73,529,724 | 0.75 (0.68–0.81) |
| South | 2009 | 509 | 74,596,130 | 0.67 (0.61–0.73) |
| South | 2010 | 545 | 75,419,767 | 0.72 (0.66–0.78) |
| South | 2011 | 520 | 76,650,163 | 0.66 (0.60–0.71) |
| South | 2012 | 563 | 77,681,893 | 0.69 (0.63–0.74) |
| South | 2013 | 550 | 78,693,557 | 0.65 (0.59–0.70) |
| South | 2014 | 509 | 79,945,474 | 0.59 (0.54–0.65) |
| South | 2015 | 579 | 81,260,812 | 0.64 (0.59–0.70) |
| South | 2016 | 595 | 82,405,493 | 0.64 (0.59–0.69) |
| South | 2017 | 646 | 83,659,214 | 0.69 (0.63–0.74) |
| South | 2018 | 707 | 84,717,024 | 0.73 (0.67–0.78) |
| South | 2019 | 764 | 85,606,773 | 0.77 (0.72–0.83) |
| South | 2020 | 889 | 86,611,804 | 0.86 (0.80–0.92) |
| South | 2021 | 961 | 86,926,028 | 0.96 (0.90–1.03) |
| South | 2022 | 1035 | 87,978,320 | 0.97 (0.92–1.04) |
| South | 2023 | 1033 | 89,219,544 | 0.98 (0.92–1.04) |
| South | 2024 | 1054 | 91,136,871 | 0.94 (0.88–1.00) |
| West | 1999 | 263 | 39,373,038 | 0.76 (0.67–0.85) |
| West | 2000 | 282 | 39,847,571 | 0.78 (0.69–0.88) |
| West | 2001 | 277 | 40,661,876 | 0.77 (0.68–0.86) |
| West | 2002 | 294 | 41,294,037 | 0.80 (0.71–0.89) |
| West | 2003 | 307 | 41,903,467 | 0.81 (0.72–0.90) |
| West | 2004 | 306 | 42,568,500 | 0.79 (0.70–0.88) |
| West | 2005 | 354 | 43,291,979 | 0.91 (0.81–1.00) |
| West | 2006 | 335 | 44,054,536 | 0.82 (0.73–0.91) |
| West | 2007 | 324 | 44,745,606 | 0.79 (0.70–0.88) |
| West | 2008 | 374 | 45,462,708 | 0.89 (0.80–0.98) |
| West | 2009 | 364 | 46,160,829 | 0.84 (0.75–0.93) |
| West | 2010 | 381 | 46,680,404 | 0.85 (0.76–0.93) |
| West | 2011 | 349 | 47,494,551 | 0.75 (0.67–0.83) |
| West | 2012 | 386 | 48,168,390 | 0.82 (0.74–0.90) |
| West | 2013 | 467 | 48,863,966 | 0.95 (0.86–1.04) |
| West | 2014 | 371 | 49,792,770 | 0.72 (0.65–0.80) |
| West | 2015 | 375 | 50,698,818 | 0.71 (0.64–0.78) |
| West | 2016 | 490 | 51,393,231 | 0.89 (0.81–0.97) |
| West | 2017 | 446 | 52,227,159 | 0.82 (0.74–0.89) |
| West | 2018 | 443 | 52,867,078 | 0.77 (0.70–0.84) |
| West | 2019 | 501 | 53,403,697 | 0.87 (0.79–0.95) |
| West | 2020 | 676 | 53,882,971 | 1.12 (1.03–1.20) |
| West | 2021 | 600 | 53,940,744 | 1.03 (0.95–1.12) |
| West | 2022 | 624 | 54,153,801 | 1.00 (0.93–1.09) |
| West | 2023 | 643 | 54,595,075 | 1.04 (0.96–1.12) |
| West | 2024 | 559 | 55,550,527 | 0.87 (0.80–0.94) |
